# Deep crustal magnetotelluric imaging of continental accretion and intracontinental deformation in central Australia

**DOI:** 10.1038/s41598-025-02830-1

**Published:** 2025-07-01

**Authors:** Graham Heinson, Ben Kay, David Baker, Relly Margiono

**Affiliations:** 1 Department of Earth Sciences, University of Adelaide, Adelaide, Australia; 2 State College of Meteorology, Climatology, and Geophysics, Tangerang, Indonesia

**Keywords:** Magnetotelluric, Central Australia, Resistivity, Conductance, Continental accretion, Orogeny, Geology, Geophysics, Tectonics

## Abstract

Central Australia preserves a record of micro-continent and craton accretion during assembly of the Paleoproterozoic Nuna supercontinent 2500 − 1600 Ma, followed by Mesoproterozoic magmatic and orogenic events that formed the Musgrave Province. The Petermann Orogeny (630 − 520 Ma) and Alice Springs Orogeny (450 − 300 Ma) resulted in north-south crustal shortening and Moho offsets up to 20 km that yield gravity variations of ~ 160 mGals. Three-dimensional inversion of 614 long-period (10–10000 s) AusLAMP MT and 36 geomagnetic depth sounding (GDS) sites spaced ~ 55 km produced resistivity estimates to 250 km depth, covering 1500 km west-east, 1300 km north-south. From 0 to 5 km, resistivity maps the extent and thickness of Neoproterozoic Officer, Amadeus, Ngalia and Georgina Basins, and Mesozoic Eromanga basin. At all crustal depths the Arunta Province and northern Musgrave Province are resistive (> 10000 Ω.m), bounded by lower crustal conductive zones (< 10 Ω.m) to the north, east and south that align with suture zones associated with Paleoproterozoic accretion of ribbon continents. Lithospheric scale faults active in the Petermann Orogen (Woodroffe Thrust) and Alice Springs Orogen (Redbank Shear Zone) align with these low-resistivity zones, and we argue that graphite from carbon burial in Paleoproterozoic sediments reduces frictional strength and enable compressive deformation to localise strain.

**Supplementary Information:**

The online version contains supplementary material available at 10.1038/s41598-025-02830-1.

## Introduction

The proto-North Australian Craton accreted ribbon micro-continents from 1860 − 1800 Ma during a period of rapid growth^1,2^. Significant suture zones marking accretion have been mapped from reflection seismics and potential fields. The Willowra Suture^1,3,4^ marks the boundary between the North Australian Craton and the Aileron and Nawa Terranes, and the Gidyea Suture^5,6^ records the collision between the eastern Numil Terrane and Mount Isa Terrane. The North Australian and West Australian Cratons joined during the 1800–1765 Ma Yapungku Orogeny^7^ to form proto-Australia. It has been suggested^1,8,9^ that prior to 1500 Ma the South Australian Craton was contiguous with the proto North Australian Craton, the Archaean Gawler Craton being accreted along the Willowra Suture, and to the east of its current location. Rifting occurred at the southern margin of the Mount Isa Inlier at 1500 Ma, followed by southward drift, rotation and finally re-accretion from 1330 to 1100 Ma to the West Australian Craton during the Albany Fraser Orogeny^10^, and to the North Australian Craton during the Musgrave Orogeny^11,12^.

The Musgrave Orogeny is characterised as an intraplate Mesoproterozoic orogenic event (1220 − 1150 Ma) associated with a Large Igneous Province, with granulite-phase high-pressure and ultra-high temperature^13,14^ lower crust that now outcrops as part of the Musgrave Province. Temperatures > 1000 °C and pressures of about 7–8 kbar produced voluminous felsic magma that were crust and mantle derived. During the Neoproterozoic and early Palaeozoic eras from 800 − 600 Ma, an expansive, multi-phase sedimentary basin spanned large regions of northern, central, and southern Australia^15^. The Neoproterozoic sequences were part of a unified intra-cratonic depositional system known as the Centralian Superbasin, contiguous with the Adelaide Superbasin in southern South Australia^16^.

The Petermann Orogeny (630 − 520 Ma) was a major intraplate crustal shortening event, defined by west-east shear zones with crustal-scale deformation, flower-type structures^17,18^ and pressures of up to 10 kbar. The Petermann Orogeny reactivated existing lithospheric-scale faults, most notably the Woodroffe Thrust, Davenport Shear Zone and Mann Fault^19,20^ to cause a series of nappes with crustal-scale basement structures, and exhumation of Musgrave Province granulite phase rocks at surface, breaking up the Centralian Basin^21^ to isolate the Officer Basin to the south.

The Alice Springs Orogeny (450 − 300 Ma) was the final major deformation event in the same north-south orientation as the Petermann Orogeny^21,22^. However, major fault reactivation was primarily in the north-dipping faults of the Redbank Shear Zone and Napperby Thrust, exhuming the Arunta Province from beneath the Centralian Basin and separating the Centralian Basin into the Amadeus, Ngalia and Georgina Basins^23–25^.

**Fig. 1:**
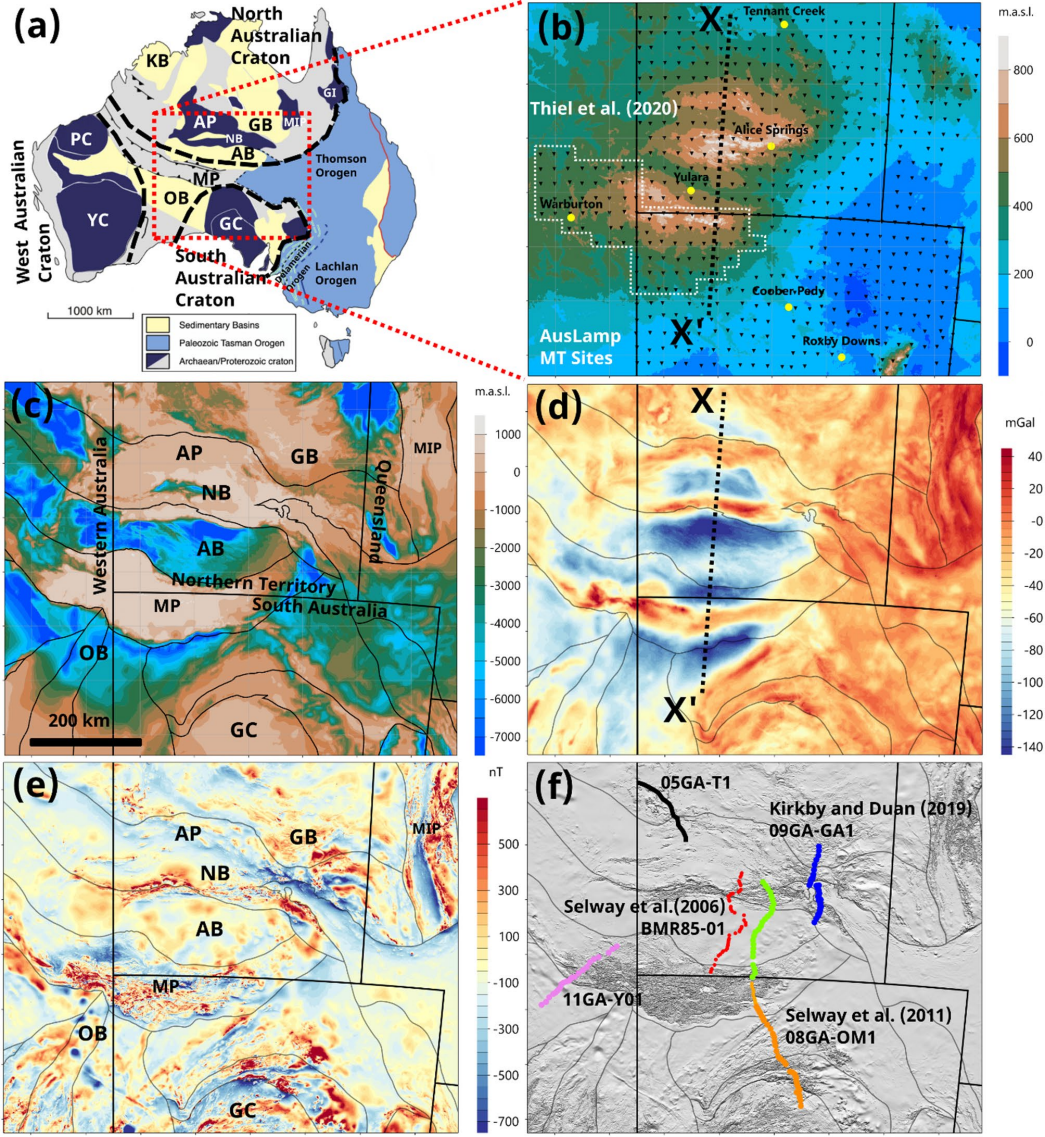
Figure 1 (**a**) Simplified map of principle geological domains of Australia, adapted Figure^27^. Thick black dashed lines show approximate extent of the West, North and South Australian Cratons; (**b**) Location of AusLAMP MT sites, State and Territory borders by thick black lines, and towns on topography, along with extent of the prior 3D MT study of Thiel et al.^28^; (**c**) Depth of Proterozoic basement with crustal domain boundaries shown by fine black lines^26^; (**d**) Bouguer anomalies; (**e**) Total magnetic intensity; (**f**) Sun-shaded total magnetic intensity with Sun-shaded total magnetic intensity with prior (red, blue and orange sites) published MT transects^12,29,30^ and reflection seismic lines BMR85-01; 05GA-T1; 08GA-OM1; 09GA-GA1; and 11GA-YO1. Cratons and Provinces: PC – Pilbara Craton; YC – Yilgarn Craton; GC – Gawler Craton; MP - Musgrave Province; AP – Arunta Province; MIP – Mount Isa Province; GI - Georgetown Inlier. Basins: OB – Officer Basin; AB – Amadeus Basin; NB – Ngalia Basin; GB – Georgina Basin; KB – Kimberley Basin; EB - Eromanga Basin. Profile X-X’ shown in in Fig. 8. Areas are shown in UTM 52 S projection. Elevation, magnetic and gravity data were sourced from Geoscience Australia’s Geophysical Archive Data Delivery System (https://portal.ga.gov.au/persona/gadds) released under a CC BY 4.0 license (https://www.ga.gov.au/copyright). Depth to Proterozoic basement were obtained from Geognostics OZ SEEBASE^®^ 2021 (https://www.geognostics.com/oz-seebase-2021) released under a CC BY 4.0 license. Figures generated using Viridien Geotools V.4.0.3.12574 (https://www.viridiengroup.com/expertise/multiphysics-imaging/geotools) and Inkscape V1.2.1 (https://inkscape.org/cs).

Shows the regional geology, geomorphology, potential field response and prior MT and seismic reflection geophysical surveys, along with the major crustal domain boundaries^26^. In Fig. 1b, the current availability of 614 AusLAMP and legacy MT sites and 36 GDS sites are shown with spacing of about ~ 55 km, spanning an area 1500 km west-east and 1300 km north-south. The topography reflects the exhumation of the Musgrave Province (MP) during the Petermann Orogeny, and the Arunta Province (AP) during the Alice Springs Orogeny, with the Neoproterozoic Officer Basin (OB), Amadeus Basin (AB), Ngalia Basin (NB) and Georgina Basin (GB) being less elevated. However, there is a pervasive upward flexure of the crust (along profile X-X’ for example) over a wavelength of about 1000 km from north to south^22^. To the east, the Mount Isa Province has slightly higher elevation, but generally the Gawler Craton (GC) to the south and Mesozoic Eromanga Basin (EB) to the east are < 200 m elevation and relatively flat. The depth to Proterozoic basement map in Fig. 1c reflects the distribution of basins and cratonic units as shown in Fig. 1a, with localised thickening of the northern margins of the Officer Basin (OB) and Amadeus Basin (AB) associated with the intracratonic orogenic events^21^.

The Bouguer map in Fig. 1d is one of the most iconic and significant geophysical signatures globally^25,31^. The profile X-X’ exhibits two scales of geometry. Firstly, over a wavelength of ~ 1000 km, there is a broad-scale negative Bouguer trend that mirrors topography and suggest that the elevation is supported by thicker crust in isostatic equilibrium. Secondly, with a north-south wavelength of 100 km or less, distinct west-east orientated anomalies of magnitude > 100 mGal are evident with a strike length of many hundreds of kilometres. Such abrupt changes were used to argue for a thick-skinned crustal deformation with Moho-penetrating faults imaged in the BMR85-01 seismic reflection profile^23,24^ that offset the Moho by 20 km or more^25^. The primary fault structures are the Woodroffe Thrust during the Petermann Orogeny and the Redbank Shear Zone during the Alice Springs Orogeny^21,22,25^. Deformational strain associated with both orogenies has been proposed to be localised to these crustal faults, implying that crust under the Amadeus Basin is lithologically strong to retain the Moho offsets for > 500 Ma^22,32^.

The total magnetic intensity (TMI) map in Fig. 1e and sunshade from the north in Fig. 1f reflect the variability of basin sedimentary cover and the exposed cratonic basements as shown in Fig. 1a. Moreover, the TMI image also provides evidence of major east-west shearing in the Musgrave Province^33,34^, and at a broader scale defines the primary tectonic elements, particularly at cratonic margins^26^, to establish a deep expression of Paleoproterozoic accretion and evidence for suture zones^1^.

Previous surveys and interpretations can be grouped into 2D broadband MT transects along reflection seismic lines^12,29,30,35^ in Fig. 1f, and an initial 3D array of long-period MT for the western and southern Musgrave Province^28^ in Fig. 1b. Although these transect surveys provided localised information and constraint on dip and dip-orientation of major crustal faults they lack the spatial aperture to provide a regional context. Similarly, the 3D model of the Musgrave Province was limited by resistivity structural changes outside the array boundaries.

In this paper, we report on new 3D inversions of the AusLAMP MT data with sufficient aperture to span the extent of the geomorphological and geophysical structures evident in Fig. 1. With a ~ 55 km inter-site spacing, lateral resolution is less than that of the prior 2D transects but provides greater spatial imaging of heterogeneity in three dimensions, and insight into the sub-lithospheric mantle.

## Results

**Fig. 2:**
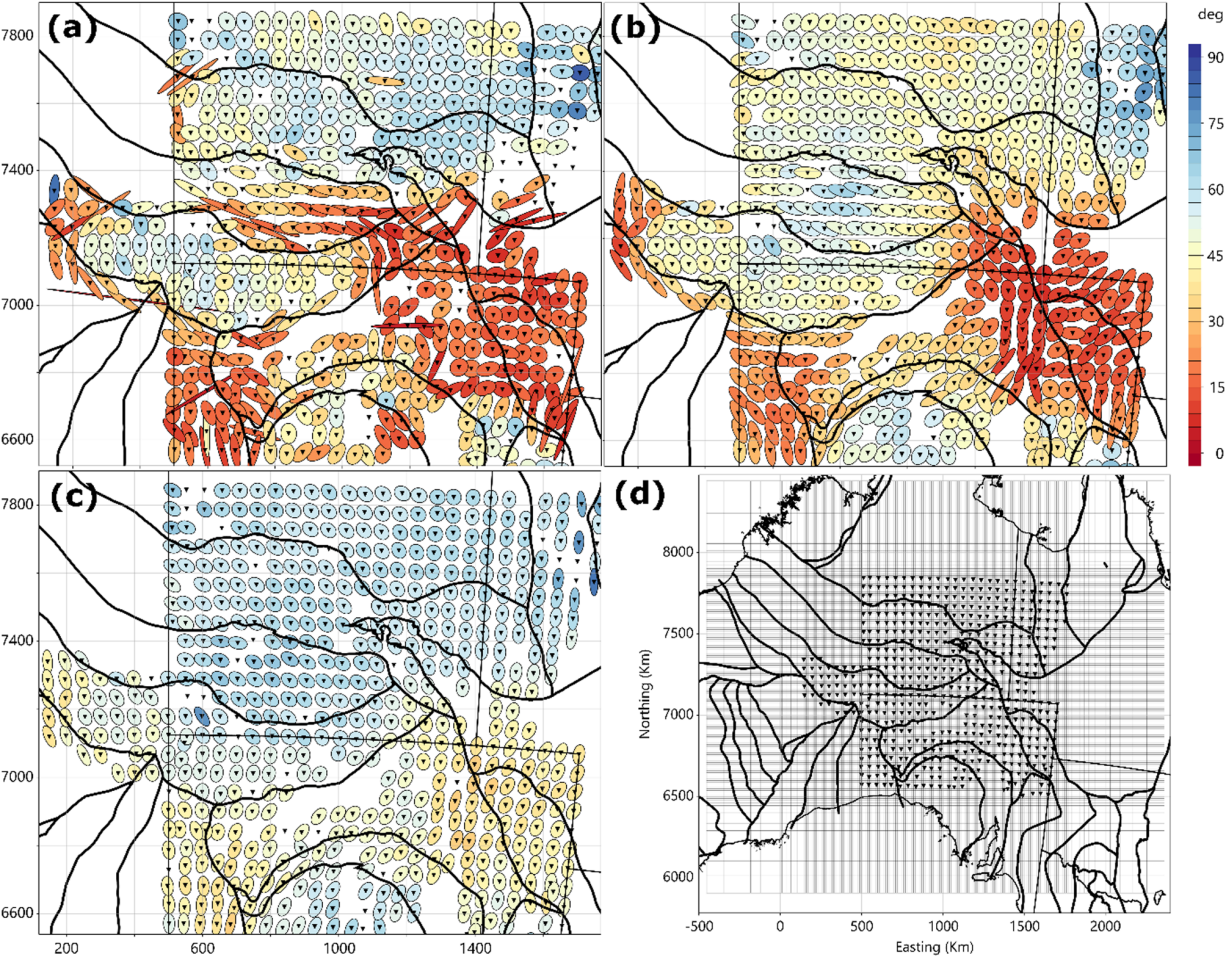
Phase tensors for periods of (**a**) 21 s, (**b**) 215 s, and (**c**) 2154 s for almost all of the MT responses, plotted with major crustal boundaries^26^. (**d**) The 3D grid used in the inversion. Areas are shown in UTM 52 S projection. Figures generated using Viridien Geotools V.4.0.3.12574 (https://www.viridiengroup.com/expertise/multiphysics-imaging/geotools) and Inkscape V1.2.1 (https://inkscape.org/cs).

Phase tensors, coloured by minimum phase are shown in Fig. 2a-c for periods of 21, 215 and 2154 s on major tectonic boundaries^26^. The minimum phase is coloured such that reds indicate that the Earth is increasingly conductive with depth, and blue shows an Earth becoming more resistive with depth. A few sites were omitted from further analyses as they displayed local three-dimensional distortion that are significantly different from neighbouring sites and thus could not be modelled with grid cells of 10 km. Data quality is generally excellent across all sites in the bandwidth of 10–10,000 s. Phase tensor ellipses define the structural trends associated with the major crustal domains and are most polarised (or elongated) on the margins of the domains, particularly at the shortest periods. Sedimentary basins are evident as low resistivity regions as defined by the minimum phase of < 45°. At periods > 1000 s, there is less heterogeneity in the responses and phase tensors vary only slightly over wavelengths of hundreds of kilometres. Three-dimensional inversion was undertaken of all MT responses on a grid shown in Fig. 2d for a bandwidth of 10–10,000 s, details are provided in the Methods section.

**Fig. 3:**
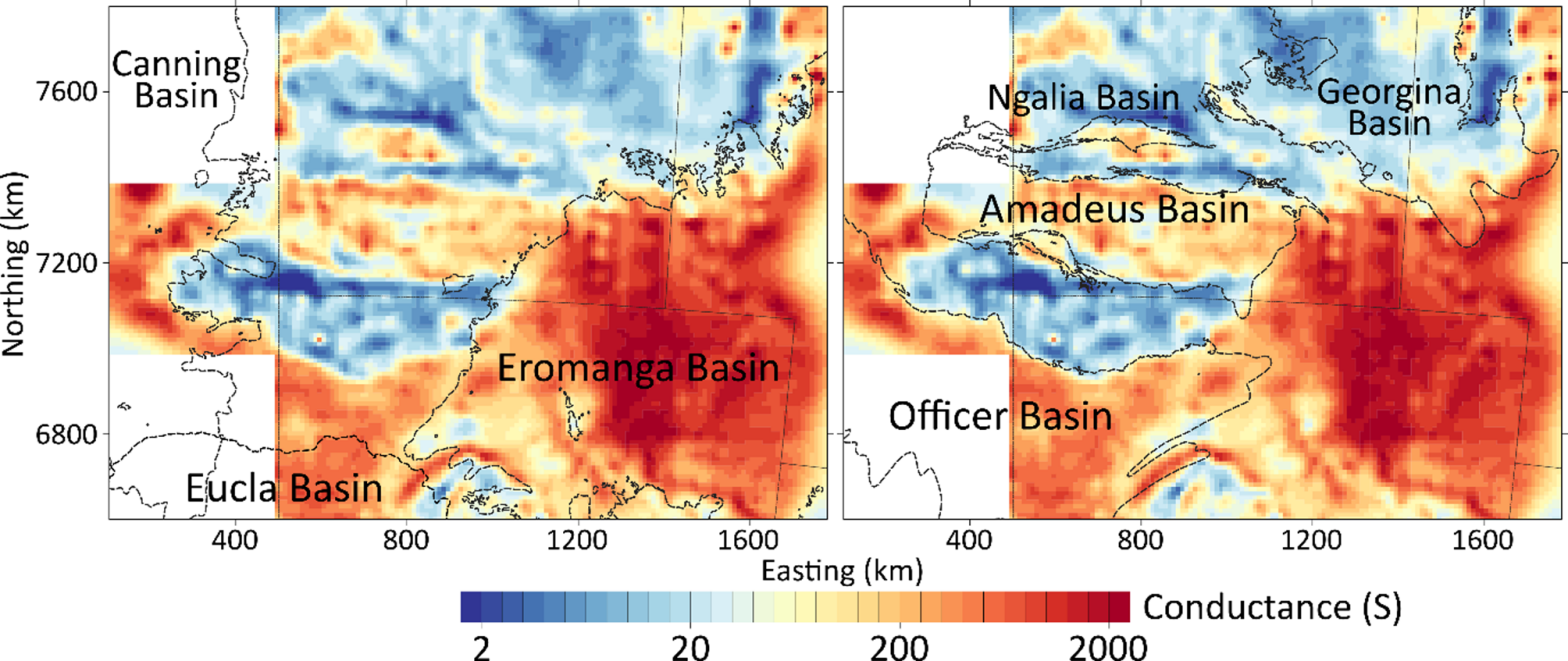
Conductance in units of Siemens for the top 5 km of the model with (left) the outline of Mesozoic basins, and (right) Neoproterozoic basins. The model is only shown for areas where AusLAMP sites are available, and unconstrained regions in Western Australia omitted. Areas are shown in UTM 52 S projection. Basin shapefiles were sourced from Geoscience Australia’s Digital Atlas of Australia (https://digital.atlas.gov.au/) and released under a CC BY 4.0 license (https://www.ga.gov.au/copyright). Figures generated using Viridien Geotools V.4.0.3.12574 (https://www.viridiengroup.com/expertise/multiphysics-imaging/geotools) and Inkscape V1.2.1 (https://inkscape.org/cs).

Three-dimension inversions of MT data, described in the Methods section, depend on model parameterisation, particularly on smoothing weight in both horizontal and vertical directions. However, it is well established that the depth-integrated conductivity (reciprocal of the modelled resistivity) is more robust to model parameter weightings in one-dimension^36^, and in the Supplementary Section Figure S1 we show that this is the case for the current model configuration in three dimensions. Figure 3 shows the model conductance for the top 5 km in units of Siemens, with Neoproterozoic and Mesozoic basins outlined. As the array in Western Australia is limited in extent, regions to the north and south of the Musgrave Province are masked, although the model grid extends in all directions and with padding cells hundreds of kilometres in Fig. 2d. To convert to an equivalent integrated resistivity, the reciprocal of the modelled conductance must be multiplied by the interval thickness; thus, a conductance of 2000 S implies an average resistivity over 5 km of 2.5 Ω.m and 2 S is equivalent to a bulk resistivity of 2500 Ω.m.

Regions of higher conductance indicate the presence of saline fluids in porous sediments and conduction in clays. By contrast, basement rocks with little cover have much lower conductance < 20 S. There is generally good correspondence between the conductance and depth to Proterozoic basement in Fig. 1c with the Georgina Basin (GB) being shallowest and hence least conductive. Such maps are useful as verification of model veracity and illustrates the intrinsic resolution for sites spaced ~ 55 km apart. The Mesozoic Eromanga Basin has much higher conductance than any of the Neoproterozoic Basins, due to thick clay layers deposited in a deep marine setting^37^.

shows four representative conductance layers for intervals 10 km thick, grouped into two upper crust layers to a depth of 25 km, and two lower crust layers to the Moho at a depth of about 45 km for this region^38^. Locations of gold and copper resources are shown relative to the lower-crustal conductance maps. In the Supplementary section Figure S1 shows similar conductance maps for the same vertical interval, with varying smoothing parameters. The equivalent average resistivity range for each interval is 5000 Ω.m (blue) to 5 Ω.m (red) on a log-scale.

**Fig. 4:**
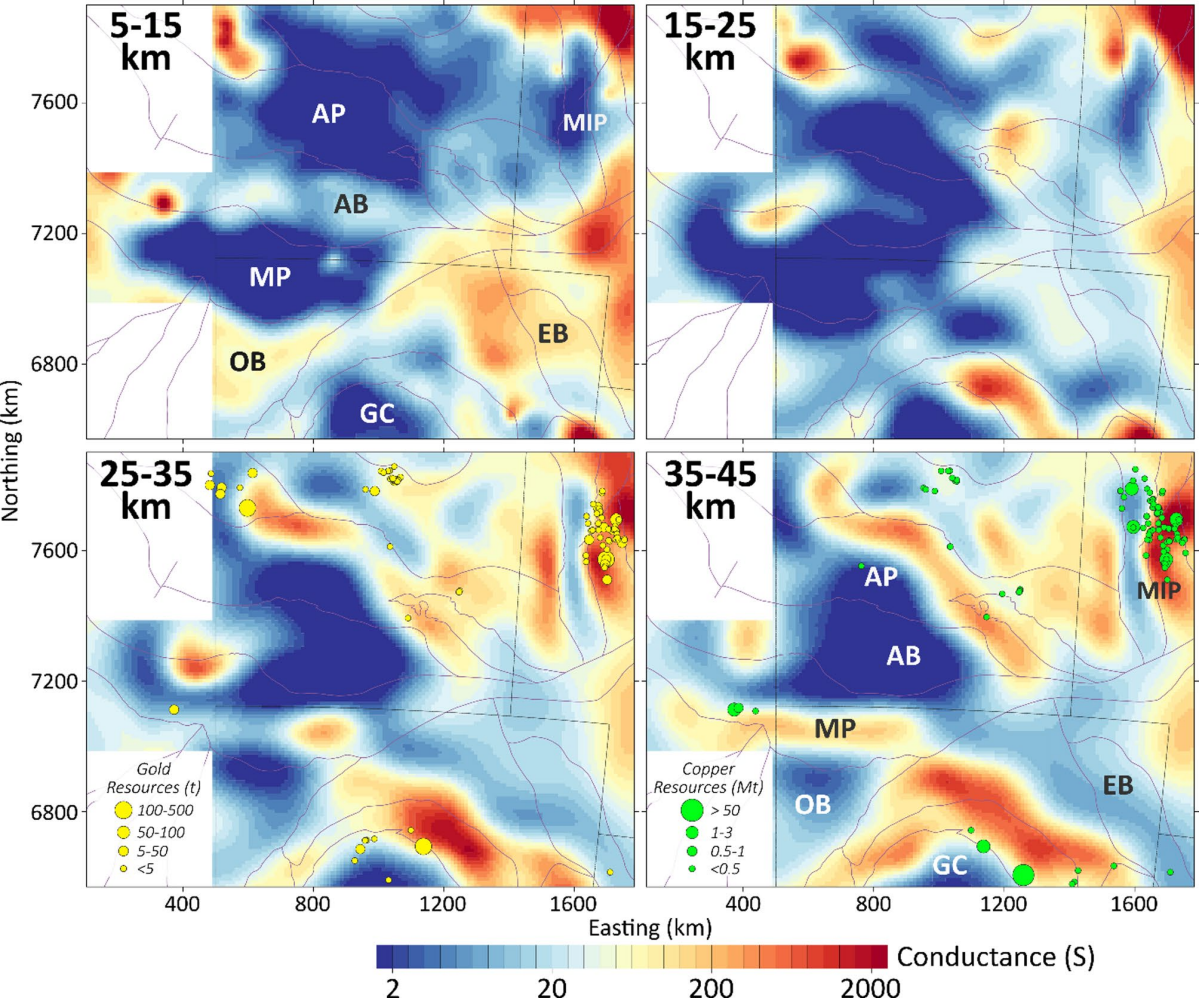
Figure 4 Conductance in units of Siemens of four crustal intervals. Gold and copper resources are shown in the lower two panels. Labels are described in Fig. 1 and the text. Areas are shown in UTM 52 S projection. Copper and gold resources were sourced from Geoscience Australia’s Digital Atlas of Australia (https://digital.atlas.gov.au/) and released under a CC BY 4.0 license (https://www.ga.gov.au/copyright). Figures generated using Viridien Geotools V.4.0.3.12574 (https://www.viridiengroup.com/expertise/multiphysics-imaging/geotools) and Inkscape V1.2.1 (https://inkscape.org/cs).

For the upper crust from 5 to 15 km, the deeper parts of the Officer Basin (OB), Amadeus Basin (AB), and Eromanga Basin (EB) are most evident. Here it is expected that sedimentary thicknesses may exceed 5 km in the northern Officer Basin (OB) and Amadeus Basin (AB) (in Fig. 1b) and the inversions will always lead to a downward smoothing of modelled resistivity beneath very low resistivity regions, even though the Eromanga Basin (EB) does not appear to be of thickness > 5 km. In areas of exposed basement in the Gawler Craton (GC), Musgrave Province (MP), Arunta Province (AP) and Mount Isa Province (MIP) the conductance is very low (~ 2 S) as would be expected. From 15 to 25 km, the conductance has a similar morphology, and to some extents smooths vertically down from the shallower part of the crust. Smaller conductors of dimensions comparable to site spacing may be artifacts, and this is probably most accentuated in areas at the edges of the AusLAMP grid.

In both lower crust 25–35 km and 35–45 km images, long and linear conductors are evident that bound a significantly resistive region (< 2 S or equivalently > 5000 Ω.m) that spans the crust under the Amadeus Basin (AB), and the southern part of the exposed Arunta Province (AP) and northern part of the Musgrave Province (MP). These lineaments have conductance of > 200 S (equivalently < 50 Ω.m) which is anomalous for crustal silicates at typical crustal temperatures < 700^o^ C^39^. Conductors are aligned with the northern tectonic unit of the Gawler Craton (GC) known as the Nawa Terrane; the southern boundary of the Musgrave Province (MP); the northern boundary of the Arunta Province (AP) in the Aileron Terrane; and the eastern and western margins of the Mount Isa Province (MIP). Such conductors are typically > 200 km long and are imaged with a width of about 50 km. We note that such width is compatible to site spacing and thus is at the limit of resolution. There are variations of conductance along these features, but all are most continuous below 35 km to at least the Moho^38^. Location of copper and gold reserve estimates in Fig. 4 are broadly located along margins of lower crustal conductors. Such spatial correlations with lower crustal resistivity have been noted elsewhere in Australia and globally^40–46^, but the causal mechanisms that link deep conduction with metal transport are not well understood^45,46^.

**Fig. 5:**
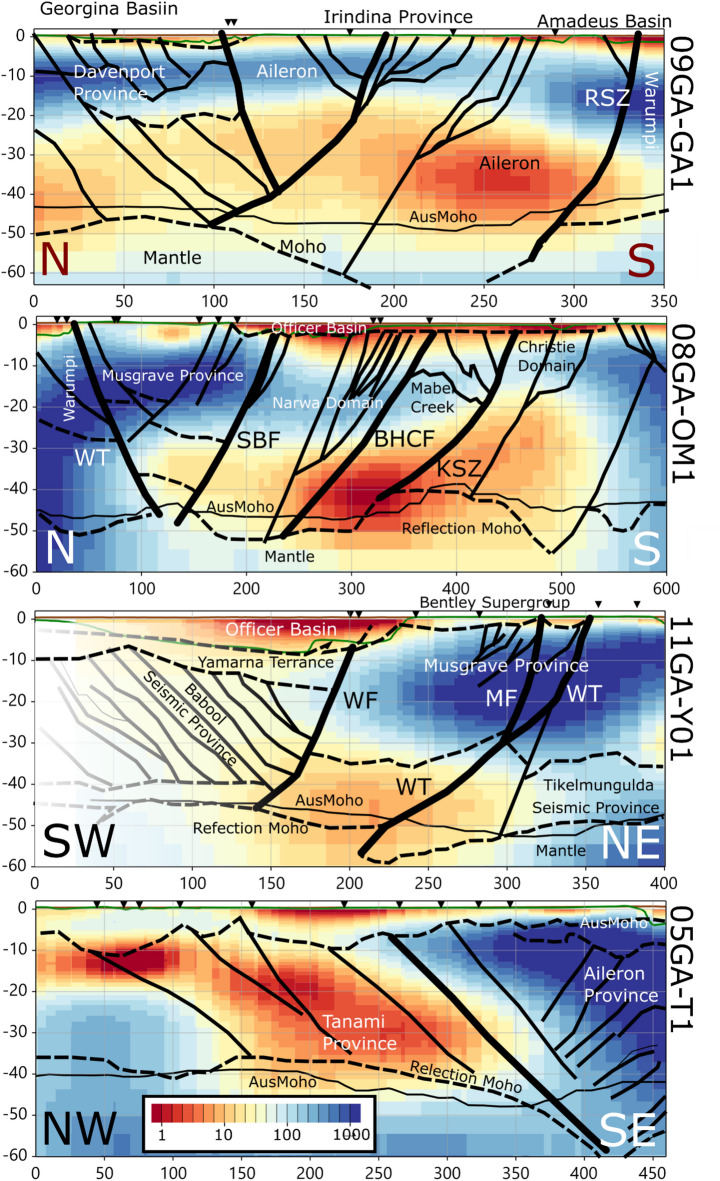
Reflection seismic profiles shown in Fig. 1f to a depth of 60 km and horizontal extent varying from 350 to 600 km. Black triangles show adjacent AusLAMP sites to provide an indication of the lateral resolution of the resistivity model. Structural and geological interpretations are shown by black lines^26^, with the most significant lithospheric-scale faults indicated with thicker lines. The interpreted seismic Moho is shown by the deepest dashed line, and the Moho depth from the AusMoho database^38^ by a finer black line. Model 11GA-YO1 is deliberately faded from 0–100 km profile range as the AusLAMP array does not extend this far. RSZ- Redbank Shear Zone; WT – Woodroffe Thrust; SBF – Sarda Bluff Fault; BHCF – Box Hole Creek fault; KSZ – Karari Shear Zone; WF – Windurra Fault; MF – Mitika Fault. Figures generated using Viridien Geotools V.4.0.3.12574 (https://www.viridiengroup.com/expertise/multiphysics-imaging/geotools) and Inkscape V1.2.1 (https://inkscape.org/cs).

The veracity of the 3D resistivity model can be tested against interpreted seismic reflection lines 05GA-T1, 08GA-OM1, 09GA-GA1 and 11GA-YO1 in Fig. 1f. Older lines BMR85-01 (in Fig. 1f) to BMR85-04 were shot in 1985 with explosives that images attributes of the Redbank Shear Zone, but these lack the fidelity and extent of reflection lines more recently collected with Vibroseis. Figure 5 shows resistivity sections along the four seismic lines with structural and geological interpretation to a depth of 60 km^26^. These lines vary in length from 350 km for 09GA-GA1 to 600 km for 08GA-OM1. Proximal AusLAMP MT stations to the lines are indicated by black triangles, and thus the 3D resistivity model lateral resolution is typically on a scale-length of 55 km compared with much higher spatial resolution from the seismic interpretations. We note that structural seismic interpretations are not definitive or unique, and that there is a degree of subjectivity in delineating fault dip, extent and significance. Figure 5 includes both the AusMoho depth from the continental-scale arrays^38,47^, and the interpreted Moho from the seismic Sect. ^26^.

In all four cross-sections in Fig. 5, the boundaries and depths of the sedimentary basins in the top 5 km are generally well defined as a layer of low (1–10 Ω.m) resistivity, and compatible with the estimate of depth to Proterozoic basement in Fig. 1c. With exception of Tanami line 05GA-T1, the upper crust to 20 km depth is resistive (> 100 Ω.m). Lower-crustal resistivities vary between 1 and 1000 Ω.m, and there appears to be some spatial correlation with the extent of most significant lithospheric faults (thick black lines).

The resistivity section for Line 09GA-GA1 was compared in more detail using broadband MT stations^30^ but lacked aperture to resolve deeper crustal attributes. The most striking feature is that the low resistivity lower-crust beneath the Aileron Terrane (northern Arunta Province) of ~ 10 Ω.m that is proximal to the inferred depth-extent of the boundary between resistive (> 100 Ω.m) Warumpi Province (southern Arunta Province) and the Aileron Terrane (denoted as the Redbank Shear Zone (RSZ) from an eastward projection).

Line 08GA-OM1 is also at the eastern margin of the Musgrave Province and the Woodroffe Thrust (WT) has a much weaker gravity signature implying smaller Moho offset. The line extends from the resistive Warumpi Province, bounded by the south-dipping Woodroffe Thrust (WT) that dips and extends to the conductive part of the lower crust. The Musgrave Province upper crust is imaged as being electrically resistive (> 1000 Ω.m). Further south along the line, the northwest dipping Karari Shear Zone (KSZ) is coincident with a very low resistivity region that has an interpreted major offset in Moho depth^26^.

The Woodroffe Thrust (WT) is also imaged along line 11GA-YO1. AusLAMP coverage is only present for half of the model section in the northern region, and hence the resistivity imaged is faded at the southern end as there is no resolution. The upper crust of the Musgrave Province is resistive, with the Woodroffe Thrust interpreted to be in the most conductive part of the lower crust that is bounded to the SW by the Windurra Fault (WF). The seismically interpreted Moho has a significant offset of about 10 km at a depths > 50 km. As for line 08GA-OM1, the Musgrave Province upper and lower crust are resistive (> 100 Ω.m) to at least the Moho.

Line 05GA-T1 separates the boundary between the Tanami Province and Aileron Terrane (northern Arunta Province), with the southeast dipping faults interpreted to be associated with the Willowra Suture. The upper crust is significantly more conductive that the other three profiles and is spatially aligned with orogenic gold deposits in the Tanami region, as shown in Fig. 4 in the 25–35 km conductance map. Paleoproterozoic suture zones have been images as being electrically conductive at shallow depths in other regions of Australia, including the Gidyea Suture^48^, Curnamona Province^49^ and Southern Gawler Craton^50^. Orogenic gold deposits in western Victoria are also spatially aligned with regions of low resistivity at depths of 20–40 km^51^.

## Discussion

**Fig. 6:**
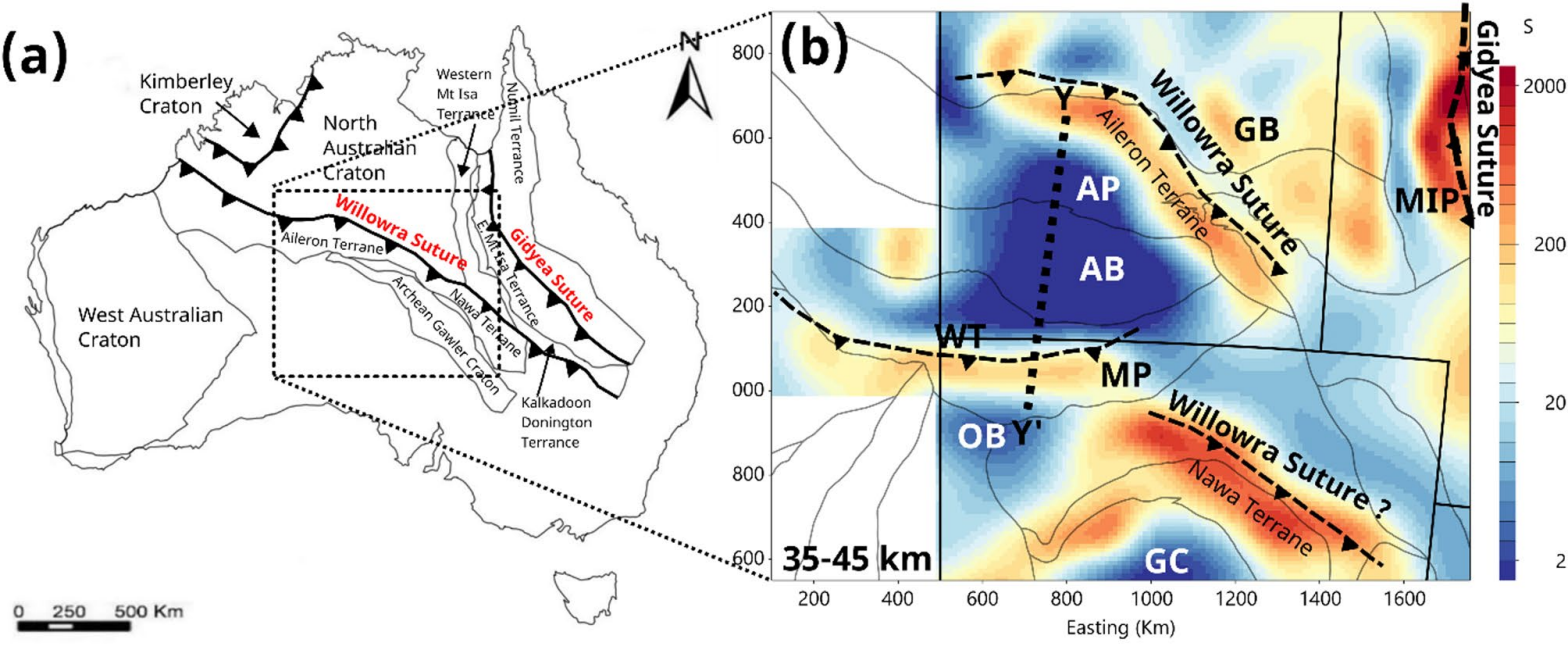
(**a**) Paleoproterozoic reconstruction of the North, West and South Australian Cratons^1^; (**b**) lower crustal (35–45 km) conductance in Siemens with inferred Paleoproterozoic suture zones (black dashes). Cratons and Provinces: GC – Gawler Craton; MP - Musgrave Province; AP – Arunta Province; MIP – Mount Isa Province. Basins: OB – Officer Basin; AB – Amadeus Basin; GB – Georgina Basin. Fault: WT – Woodroffe Thrust. Areas are shown in UTM 52 S projection. Figures generated using Viridien Geotools V.4.0.3.12574 (https://www.viridiengroup.com/expertise/multiphysics-imaging/geotools) and Inkscape V1.2.1 (https://inkscape.org/cs).

The primary insight from the 3D model of central Australia is of low-resistivity (> 200 S, < 50 Ω.m) lineaments at lower-crustal depths, and a very high resistivity (< 2 S, > 5000 Ω.m) crustal core beneath the northern Musgrave Province (MP), Amadeus Basin (AB), and southern Arunta Province (AP). Figure 6 shows a Paleoproterozoic reconstruction around 1800 Ma of the North Australian Craton and West Australian Craton^1^; although other plate reconstructions exist^52^ they share similar traits. A primary feature of these models^1,52^ is a long accretionary boundary that separates the proto-North Australian Craton from the Aileron Terrane that forms the northern component of the Arunta Province (AP). In Fig. 6a, the accretionary boundary is denoted the Willowra Suture^1,3,4^, extending from northwest of current Australia to the southern margin of the Western Mt Isa Terrane and Kalkadoon-Donnington Terrane, and with length > 3000 km. Other notable sutures include the Gidyea^1,5,6^ between the eastern Mount Isa Terrane, and the Halls Creek Suture^1^ that connects the Kimberley Craton with the North Australian Craton.

Paleoproterozoic sutures are noted as having a distinct resistivity signature, with long-linear conductors observed in many locations in Australia and globally^5,49,50,53^. The conductive structures are not the suture zone specifically but are proximal in the direction of accretionary convergence, and have been associated with deposition of thick black shales in foreland basin settings^53^. Around the time of the Great Oxidation Event from 2400 − 2000 Ma there was significant sequestration of organic carbon in sediments, known as the Lomagundi-Jatuli Event^46,54–57^. In the accretionary system, deep burial of carbon-rich sediments are metamorphosed as flake graphite at amphibolite to granulite facies at depths > 25 km and temperatures > 550^o^ C^46^. Although it has been argued from hand samples that connections of graphite deposited on grain boundaries are unstable at lower crustal temperatures^58^, there is evidence on a lower-crustal scale of tens to hundreds of kilometres for sufficient connections to significantly reduce overall resistivity^59^. Additionally, in some locations, lower crustal conductors can be mapped to near surface graphite deposits in areas that have exhumation of lower crust^49,50^.

Figure 6b shows conductance of the lower crust (35–45 km) and the association with Paleoproterozoic sutures. The northern border of the Nawa Terrane to the northeast of the Gawler Craton (GC) may effectively be the continuation of the Willowra Suture conductor in the North Australian Craton, after the South Australian Craton rifted at 1500 Ma, and re-accreted in the Musgrave Orogen^1^. The conductor to the west of the Gidyea Suture zone is known as the Carpentaria Conductor^5,60^. There appears to be an additional conductor parallel to the margin of the Western Mount Isa Terrane, which is perhaps an accretionary zone that has not been imaged by prior seismic reflection lines.

The conductor under the southern part of the Musgrave Province has a less obvious explanation given that the Musgrave Orogeny occurred at 1220 − 1150 Ma, much later than the Lomagundi-Jatuli Event at 2200–2000 Ma, and thus significantly less carbon was buried in sediments. The conductor lies south of the surface location of the Woodroffe Thrust that is the main zone of deformation in the Petermann Orogen, but which probably existed as a lithospheric weakness prior^61^. However, we do not have simple explanation for the conductance at this location.

**Fig. 7:**
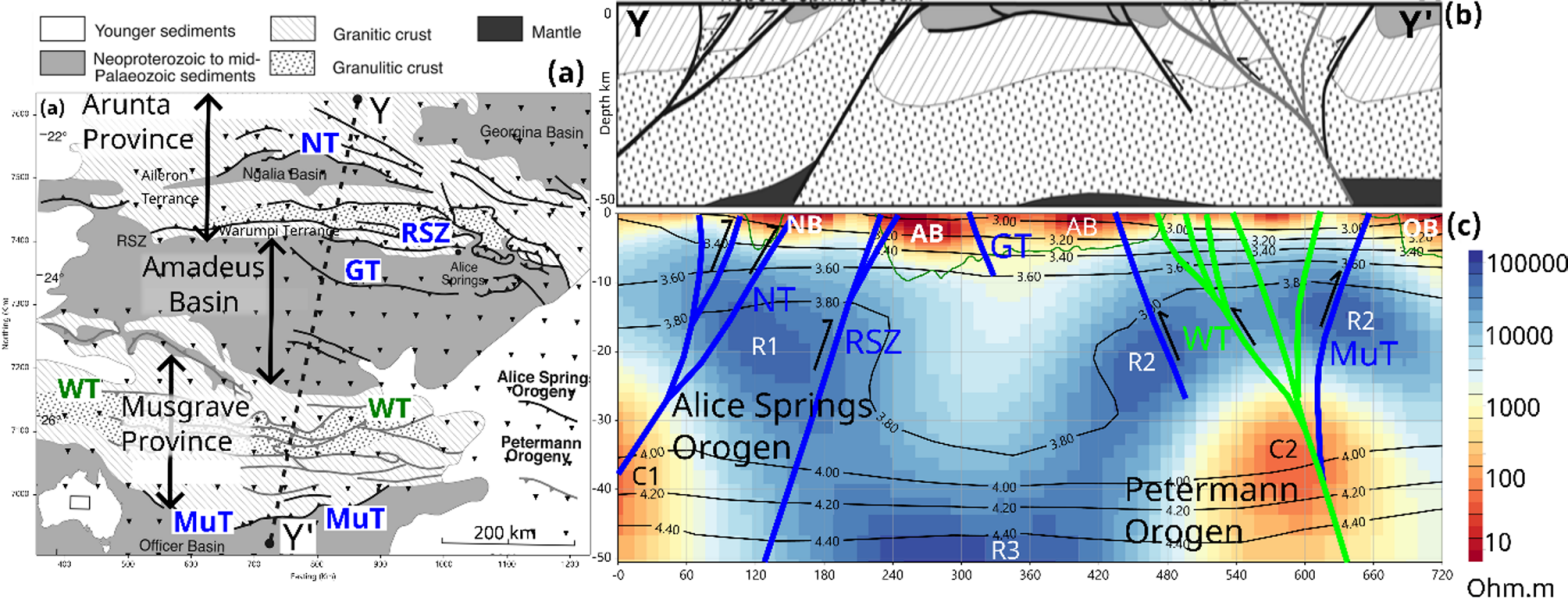
(**a**) Geological and tectonic framework of central Australia^22^, and the right hand figures are for profile Y-Y’ of (**b**) a simplified geological cross-section, and (**c**) a geophysical cross-section from the 3D resistivity model (colour scale), and shear-wave velocity contours in kilometres/s from an Australian crustal-scale model^47^. Estimated lithospheric scale faults^22^ from the geological cross-section in (**b**) are shown in green for the Petermann Orogeny, and blue for the Alice Springs Orogeny. Basins: OB – Officer Basin; AB – Amadeus Basin; NB – Ngalia Basin. Faults: WT – Woodroffe Thrust; RSZ – Redbank Shear Zone; NT – Napperby Thrust; MuT – Munyari Thrust; GT – Gardiner Thrust. The velocity model was obtained from CSIRO (10.25919/m2a2-qb97) released under a CC BY 4.0 license (https://research.csiro.au/dap/licences/csiro-data-licence/). Figures generated using Viridien Geotools V.4.0.3.12574 (https://www.viridiengroup.com/expertise/multiphysics-imaging/geotools) and Inkscape V1.2.1 (https://inkscape.org/cs).

Figures 7a and b show the geological framework and major boundary faults of the Munyari Thrust (MuT), Woodroffe Thrust (WT), Redbank Shear Zone (RSZ) and Napperby Thrust (NT) and principle geological domains^22^. It should be noted that the cross-section in Fig. 7b is a cartoon based on the BMR1985-01 profile^62^ and Bouguer anomaly data (Fig. 1d) to a depth of 50 km, but is not definitive.

Along this profile Y-Y’ the Petermann Orogen (green lines) is centred on the Woodroffe Thrust (WT), whereas the Alice Springs Orogen (blue lines) shows thick-skinned deformation with the Redbank Shear Zone (RSZ) and to a lesser extent the Napperby Thrust (NT) and Munyari Thrust (MuT). The Ngalia Basin (NB), Amadeus Basin (AB) and Officer Basin (OB) are thickened from their original Centralian Basin thickness in response to the surface uplift and thrust faulting. By comparison, the crust beneath Amadeus Basin (AB) and the Musgrave Province (MP) between the Woodroffe Thrust (WT) and Redbank Shear Zone (RSZ) is largely contiguous with few if any crustal-scale faults. In Fig. 7b the Moho is suggested^22^ to be offset at the Woodroffe Thrust (WT) and Redbank Shear Zone (RSZ) by up to 20 km, exhuming granulite-facies lower crust to upper crustal setting and surface exposure.

Figure 7c shows the same crustal-scale section to 50 km depth along Y-Y’ plotted from the 3D resistivity model, and the most recent Australian shear-wave tomography model^47^. Basins are reasonably well delineated as being low resistivity (< 10 Ω.m) due to porous sedimentary stratigraphy. Resistive regions (> 20000 Ω.m) of the crust (R1 and R2) at depths of 10–20 km are correlated with granulite-facies basement exhumed to upper crustal levels, and similarly with high shear velocity (> 3700–3900 m/s) at the same depths. Beneath the Amadeus Basin the resistivity is lower (2000–5000 Ω.m), more typical of felsic upper crustal values, and the shear-wave velocity is about 100 m/s slower than crust to the north and south. Localised offsets in the Moho are not resolved from the seismic model with 50 km cells, and the estimated depth is continuous, being thickest under the Amadeus Basin and shallowing to the north and south. However, the resistivity of the lower crust from 35 to 45 km is heterogeneous, varying from ~ 100 Ω.m in C1 and C2, but > 20,000 Ω.m in R3 for the lower crust and uppermost mantle.

Although the direction of dip of the thrust faults is interpreted from reflection seismic profiles^26,63^ the actual dip is less constrained. We note in Fig. 7c there is a spatial correlation between the inferred deep crustal extent of the thrust faults and the low-resistivity regions. It has been suggested^32,57^ that if C1 and C2 represent deeply buried carbon-rich Paleoproterozoic sediments in accretionary settings or carbon evolved from the mantle, then graphite may localise the ductile response to the north-south orientated compressional crustal stress. Deformation will thus be localised along the lithospheric-scale Woodroffe Thrust and Napperby Thrust in a thick-skinned tectonic framework. By contrast, the resistive region R3 is compatible with conduction in silicate minerals at lower crustal temperatures^39^.

**Fig. 8:**
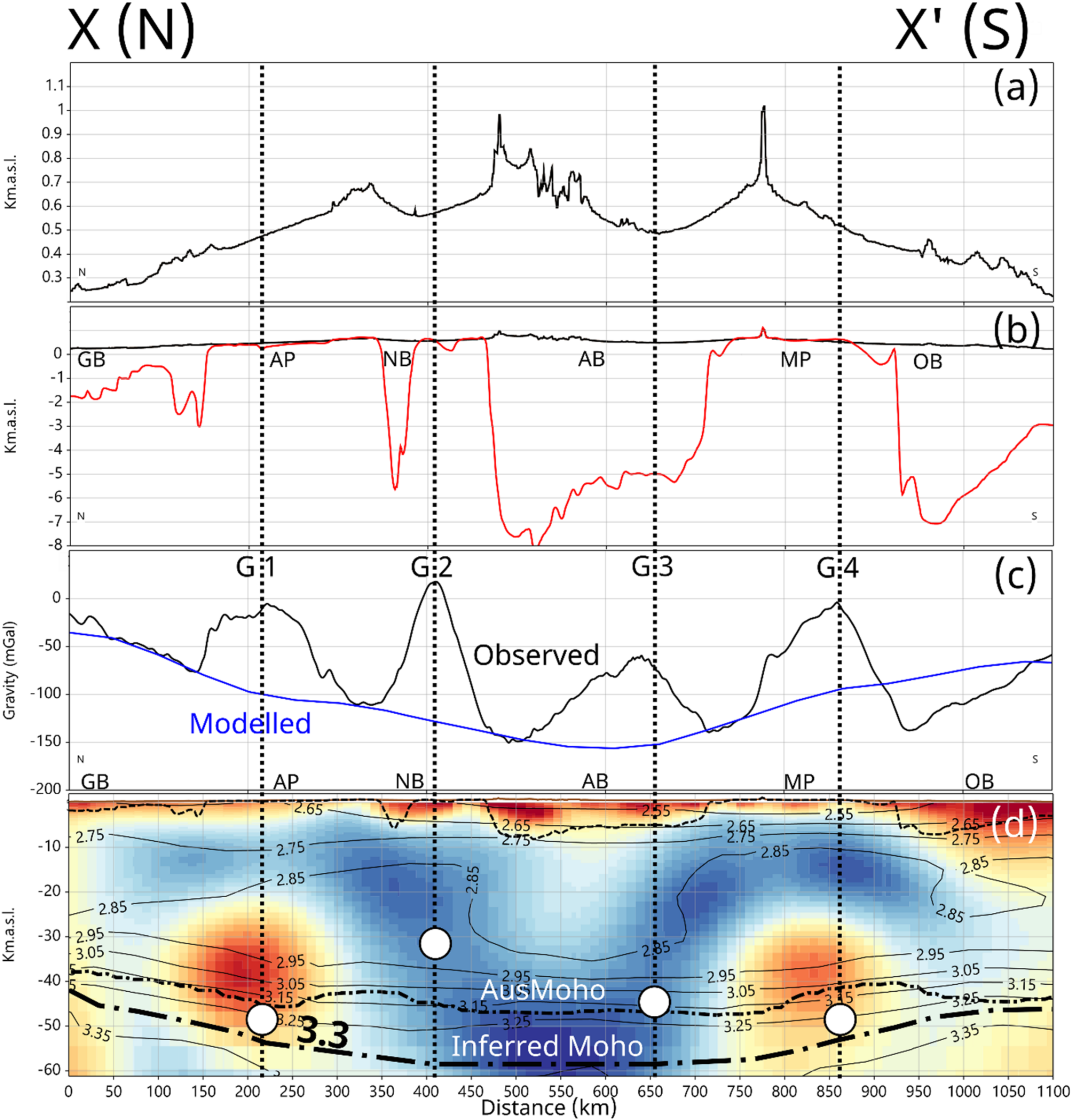
Cross sections for profile X-X’ in Fig. 1. (**a**) Elevation; (**b**) depth to Proterozoic basement; (**c**) Bouguer gravity (black line) and modelled (blue line); (**d**) resistivity from 3D model, density (in g/cm^3^) derived from regional shear-wave velocity model^47^. Also shown are: thick black dashed line as an inferred Moho defined by the 3.3 g/cm^3^ density; medium black dashed line AusMoho^38^; thin black dashed line depth to Proterozoic basement; white dots are depths to idealised line-source for gravity anomalies G1-G4. Cratons and Provinces: MP - Musgrave Province; AP – Arunta Province. Basins: OB – Officer Basin; AB – Amadeus Basin; GB – Georgina Basin; NB – Ngalia Basin. Elevation and gravity data were sourced from Geoscience Australia’s Geophysical Archive Data Delivery System (https://portal.ga.gov.au/persona/gadds) released under a CC BY 4.0 license (https://www.ga.gov.au/copyright). The density model was derived from the velocity model (10.25919/m2a2-qb97) released under a CC BY 4.0 license (https://research.csiro.au/dap/licences/csiro-data-licence/). Depth to Proterozoic basement were obtained from Geognostics OZ SEEBASE^®^ 2021 (https://www.geognostics.com/oz-seebase-2021) released under a CC BY 4.0 license. Figures generated using Viridien Geotools V.4.0.3.12574 (https://www.viridiengroup.com/expertise/multiphysics-imaging/geotools) and Inkscape V1.2.1 (https://inkscape.org/cs).

Previous studies have also linked the pronounced Bouguer anomalies in Fig. 1d with lithospheric-scale Woodroffe Thrust and Red Bank Shear Zone that offset the Moho by up to 20 km^17,25^. In Fig. 8 we show (a) elevation, (b) depth to Proterozoic basement, (c) gravity, and (d) resistivity and crustal density for the profile X-X’ in Fig. 1b and d. Profile Y-Y’ in Fig. 7 is aligned, but a shorter section. The profile is of length 1100 km and intersects the major elevation, basins and gravity lineaments approximately at right angles. Resistivity values were extracted from the preferred 3D inversion, and the densities were obtained from the regional shear-wave velocity model^47^ using empirical relationships between shear-wave velocity, compressional-wave velocity and density for crustal media^64^. Density values, in g/cm^3^ are shown with a lateral resolution of ~ 50 km, along with the Australian Moho depth^38^ and the depth to Proterozoic basement.

The long-wavelength (~ 1000 km) elevation change (0.1–1 km above sea level in Fig. 8a) and gravity anomaly (~ 160 mGals in Fig. 8c) from north to south is reflected in the crustal density variations in Fig. 8d. For example, the lower-crustal 2.95 g/cm^3^ contour is ~ 30 km depth in the north, gradually increases to 40 km beneath the Amadeus Basin (AB), and then shallows to ~ 30 km in the south. Similarly, a sub-Moho upper mantle density of 3.3 g/cm^3^ (denoted inferred Moho in Fig. 8d) is about 45 km in the northern and southern ends of the profile but is up to 60 km beneath the Amadeus Basin (AB). In the mid-crust from 10 to 30 km, beneath the Amadeus Basin there is a defined reduction in density of about 0.1 g/cm^3^.

The 3D density distribution derived from the reference shear-wave velocity model to a depth of 60 km was forward modelled^65^, details provided in the Methods section. Given the discretisation of the density model, basins in Fig. 8b were not additionally included as they are difficult to parameterise accurately. The forward response is shown as a blue line in Fig. 8c, adjusted in terms of baseline and scaled by a factor of 0.75 to match the long-wavelength characteristics of the observed data. The slightly larger dynamic range of the model (~ 210 mGals) compared to the observations (~ 160 mGals) can be accounted for by the significant number of assumptions implicit in the density model. However, the long-wavelength baseline is a good match to the observations. The largest misfit is at the northern edge of the Officer Basin where the effect of sediment thickness may explain the difference.

The long-wavelength gravity anomaly requires the model extending to 60 km (which is the extent of the shear-wave velocity model^47^. Truncating the density model with a Moho at 40–50 km results in a smaller gravity anomaly, but the gravity response is dominated by the lower density region beneath the Amadeus Basin of wavelength ~ 300 km. Thus, the regional gravity appears to require a significant thickening of the crust which supports the regional elevation. A similar crustal thickening from the northern margin of the Arunta to the southern margin of the Musgraves was also interpreted along the four seismic lines in Fig. 5., with depths to Moho extending up to 60 km.

Superimposed on the long-wavelength (~ 1000 km) gravity low are four short-wavelength (< 100 km) gravity highs denoted in Fig. 8c as G1 – G4, that have west-east strike-length of hundreds of kilometres, as shown in Fig. 1d. Relative to the regional trends, anomalies are of magnitude 100–150 mGals, and do not have obvious associations with the elevation or basin boundaries.

Interpretation of these gravity anomalies in terms of crustal density distribution is non-unique and there is no comparable reflection seismics constraints^17^. A very simple interpretation can be made with a horizontal west-east aligned line-source of anomalous density with its central axis at depth^66^. It can easily be shown that the half-width or the anomaly at half of its maximum magnitude relative to the regional baseline is equal to the depth of the centre of the cylinder producing the anomaly. Using this approach, the depths of a line-source map to ~ 45–50 km for G1, G3 and G4, and ~ 30 km for G2. For G1 and G4, such depths are effectively at the Moho and are comparable in location but slightly deeper than the conductive regions C1 and C2. By comparison the source depths for G2 and G3 lie in the lower crust based on the overall deepening of the Moho.

We therefore suggest that gravity anomalies G1 and G4 are due to significant Moho offsets that are localised due to ductile motion associated with conductive, graphitic regions. Such Moho offsets are not resolved in the shear-wave velocity model but are interpreted in the reflection seismic profiles. These anomalies are primarily associated with the Alice Springs Orogeny (G1) and the Petermann Orogeny (G4). Gravity anomalies G2 and G3 are approximately aligned with the 2.85 g/cm^3^ density contour in the upper and lower crustal. The crustal lower-density region beneath the Amadeus Basin is probably felsic, associated with voluminous melting during the Musgrave Orogeny. We speculate that G2 and G3 are thus due to thrust faulting in brittle crust defined by a rheological boundary^67^. Due to extensive melting during the Musgrave Orogeny, the crust is dry and therefore not electrically conducting.

## Conclusion

Assembly of the Paleoproterozoic Nuna supercontinent 2500 − 1600 Ma, followed by Mesoproterozoic magmatic and orogenic events that formed the Musgrave Province and subsequent Petermann Orogeny (630 − 520 Ma) and Alice Springs Orogeny (450 − 300 Ma) have left a distinct geophysical expression in terms of gravity, shear-wave velocity and electrical resistivity that have a mid and lower-crustal origin. We suggest that some if not all the lower-crustal electrical conductance can be attributed to deeply buried carbon-rich Paleoproterozoic sediments either in foreland basins or subduction settings that give rise to graphite at lower-crustal metamorphic facies. The occurrence of graphite in the lower crust may localise strain along major lithospheric faults during the subsequent Petermann and Alice Springs Orogenies. The deep conducting regions are also spatially correlated with significant copper and gold reserve estimates suggesting that they also localise the source and transport of metal ions.

## Methods

A database of 614 long-period (10–10000 s) MT and 36 GDS responses was established from a combination of new AuLAMP sites^5,28,42,68–72^ and legacy data^60,73^ as shown in Fig. 1a. The sites cover an area of 1500 km by 1300 km, with a typical site spacing of 55 km.

Long-period MT sites were collected at 10 Hz for between 4 and 6 weeks, with either AuScope and Geoscience Australia LEMI 424 instruments with fluxgate sensors and two 50 to 100 m dipoles with Ag-AgCl electrodes, or with AuScope EarthData Loggers and Bartington Mag-03 fluxgate sensors, also with two 50 m dipoles and Ag-AgCl of Pb-PbCl electrodes. Geomagnetic depth soundings were made using magnetometers developed at Flinders University that recorded for several months at 1 min sample interval and a resolution of 1 nT^60^. Long-period MT and GDS response estimates in the frequency domain were obtained using the BIRRPS processing codes^74,75^. Additional data processing and editing were carried out using MTPy^76^ and Viridien Geotools V.4.0.3.12574 (https://www.viridiengroup.com/expertise/multiphysics-imaging/geotools) .

Three dimensional inversions were carried out using Viridien Geotools V.4.0.3.12574 (https://www.viridiengroup.com/expertise/multiphysics-imaging/geotools) software based on the finite-difference forward modelling and a nonlinear conjugate gradient inversion algorithm^77,78^. The key gridding parameters were a 10 km inner grid covering all sites in Fig. 2d, with padding extending 500 km in each direction with a 20% increase in grid size with adjacent padding cells. Vertically, the top layer above sea level was 25 m thick with no topography, increasing by 5% per cell with depth to 50 km just below Moho depth, and then at 20% per cell with depth to 1000 km. There were 191 cells west-east, 163 cells north-south, and 86 cells vertically to give a total of ~ 2.7 million cells in total.

The start model was 100 Ω.m to 410 km depth, 10 Ω.m from 410 to 670 km, and 1 Ω.m below 670 km. The deeper mantle resistivities were included based on robust global satellite-derived models^79^ and have the additional benefit of satisfying boundary conditions of negligibly small induced fields that are uniform below the transition zone.

The inversion used 16 periods, spaced evenly at 5 per decade from 10 s to 10,000 s. Error floors were set at 5% on each of the four impedance components, and a fixed error floor of 0.02 for the vertical field. The inversion included an estimate of site-by-site local distortion, but a high weighting was used to allow the inversion to include variability mostly as resistivity in the subsurface. Smoothing parameters were biased to vertical variations over horizontal as inter-site spacing was typically ~ 55 km, but vertically the resistivity varies more significantly over scale-lengths of one kilometre or less near the surface. Horizontal smoothing (tauH) and vertical smoothing (tauV) were varied between 0.01 and 1; our optimal model has tauH = 1 and tauV = 0.1. Near-surface smoothing to a depth of 500 m was included to ensure that modelled resistivities near the surface were uniform across the mesh, based on the premise that near-surface sedimentary layers are laterally extensive and continuous between sites.

Overall, the misfit with tauH = 1 and tauV = 0.1 had an RMS of 1.6 which was deemed to be the preferred model. Model fits for apparent resistivity invariant and phase invariant are shown in the Supplementary Section Figure S2 for periods of 21 s, 215 s and 2154 s; fits of the tipper magnitudes are also shown in Figure S3 for periods of 215 s and 2154 s. Layer conductance models are shown in Supplementary Section Figure S1 for smoothing parameters tauH and tauV ranging from 1 to 0.01.

We primarily focus on insights gained for the crust and uppermost mantle. However, the bandwidth of MT responses provides further constraints into the deeper lithosphere and asthenosphere structure. Using the preferred 3D inversion, and noting that conductance slices are robust to the model parameterisation, in the Supplementary Section and Figure S4 we discuss broad implications from the conductance of the sub-Moho lithosphere (50–150 km) and asthenosphere (150–250 km), based on an estimated lithospheric thickness of about 150 km^80^.

Gravity forward modelling was undertaken with the open-source gravimetric forward modelling (GFM) software package for Matlab^65^. The software can be accessed with the link https://github.com/cwjwhu/Gravity-forward-modeling-GFM-. The density model was derived from the Australian shear-wave velocity model^47^ and discretised in blocks 50 km by 50 km wide, and 5 km thick. The density model covered a similar area extent as the MT sites shown in Fig. 1b.

## Electronic supplementary material

Below is the link to the electronic supplementary material.

Supplementary Material 1

## Data Availability

All MT and GDS data are available from the Geoscience Australia (https://ecat.ga.gov.au/geonetwork), the State Government of South Australia SARIG (https://map.sarig.sa.gov.au/) and National Computational Infrastructure (NCI).(https://www.nci.org.au/).
