# Supplementary material for: Deep crustal magnetotelluric imaging of continental accretion and intracontinental deformation in central Australia
Source: Sci Rep. 2025 Jul 1;15:22008. doi: 10.1038/s41598-025-02830-1 (PMC12214958; doi:10.1038/s41598-025-02830-1)
Supplement: Supplementary file 1 — Supplementary Material 1 [file 41598_2025_2830_MOESM1_ESM.docx]

**Deep crustal magnetotelluric imaging of continental accretion and intracontinental defamation in central Australia**

**Supplementary Section**

Graham Heinson^1^*, Ben Kay^1^, David Baker^1^ and Relly Margiono^1,2^

^1^Department of Earth Sciences, University of Adelaide, Adelaide, Australia

^2^ State College of Meteorology, Climatology, and Geophysics, Tangerang, Indonesia

*Graham.Heinson@adelaide.edu.au

**Smoothing Parameters**

Several 3D inversions were run to determine an optimal combination of model smoothing parameters and the sensitivity of the model. Smoothing parameters in the horizontal direction (tauH) and vertical direction (tauV) were varies between 1 and 0.01, on the basis that smaller values allow the model to be rougher in the respective dimensions and fit the data to smaller RMS. Three model outputs are shown in Figure S1 as conductance maps over four intervals of the crust. Each conductance map is the depth-integrated conductivity over a 10 km interval, for the upper crust (5-15 km, 15-25 km) and lower crust (25-35 km, 35-45 km). The models with tauH = tauV = 1 had an RMS of 1.8; tauH = TauV = 0.1 had an RMS of 1.3; and tauH = tauV = 0.01 had an RMS of 1.2. Other combinations of smoothing are, of course, possible, but it was useful to explore the primary differences between the smoothest and roughest exemplars.

Figure S1 shows that the model conductance maps become rougher with less smoothing constraint, however the primary features are quite robust. Models become more complex for only small improvement in overall RMS fit, but geologically there is little difference in the insights gained from the models.


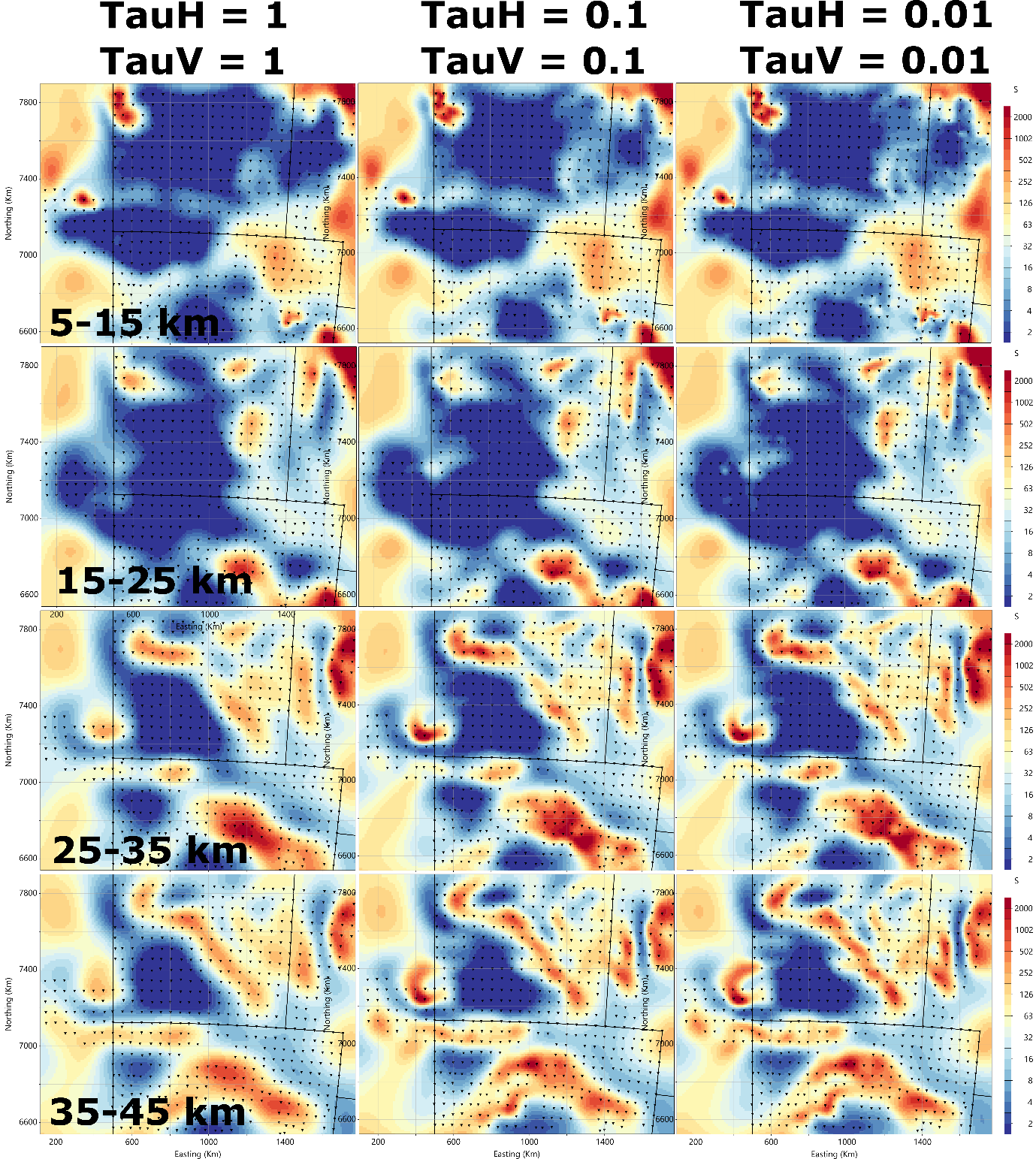


Figure S1: Conductance slices for 10 km intervals of the crust for three sets of model smoothing parameters. Areas are shown in UTM 52S projection. Figures generated using Viridien Geotools V.4.0.3.12574 (<https://www.viridiengroup.com/expertise/multiphysics-imaging/geotools>) and Inkscape V1.2.1 (<https://inkscape.org/cs>).

**Model Fits**

The optimal 3D model is described in the Methods section of the main paper, and the optimal smoothing parameters were tauH = 1 and tauV = 0.1. Such smoothing parameters effectively allow the model to vary a factor of ten more in the vertical direction compared to the horizontal. Overall, the model fits the entire data set of 609 sites used in the inversion with 8963 data-points to an RMS of 1.65. The inversion fits all components of the impedance tensor, but rather than show fits to each component separately it is more instructive to show fits to the impedance invariant (defined here as the determinant of the impedance tensor). Such invariant impedance which is a complex number can be represented by the invariant apparent resistivity and the phase.

Figure S2 shows the normalised fits to invariant apparent resistivity and phase. The normalisation is, for apparent resistivity in Figures S1a,c,e, the ratio of the observed to the modelled response. In this case, a factor of 2 implies that the observed is twice the value of the modelled, and the converse of the modelled being twice the observed apparent resistivity is shown by a factor of 0.5. A factor of 1 implies that the modelled and observed data are the same. Normalisation for the phase is simply the difference between the observed and modelled phase.


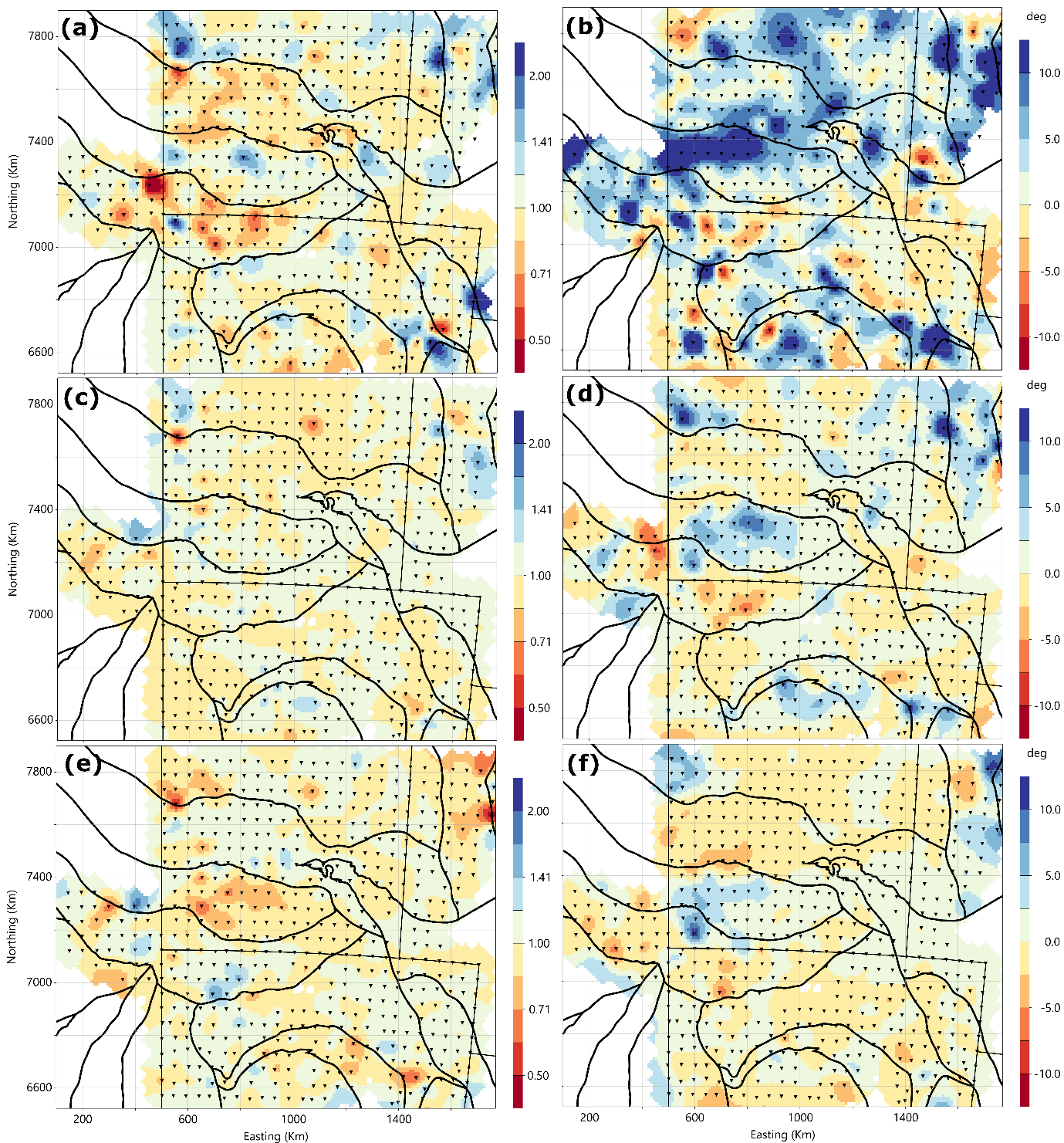


Figure S2: Normalised invariant apparent resistivity (observed/modelled) for periods of (a) 21 s; (c) 215 s; (e) 2154 s. Normalised invariant phase (observed – modelled) for periods of (b) 21 s; (d) 215 s; (f) 2154 s. Areas are shown in UTM 52S projection. Figures generated using Viridien Geotools V.4.0.3.12574 (<https://www.viridiengroup.com/expertise/multiphysics-imaging/geotools>) and Inkscape V1.2.1 (<https://inkscape.org/cs>).

The inversion has error floors set at 5% of the impedances, which is therefore 10% in apparent resistivity and about 2.9 degrees in phase. Based on this error floor and an RMS misfit of 1.65, it would be expected that the data fit will be in colour ranges immediately above and below a ratio of 1 (green to yellow) for apparent resistivity and in the colour ranges immediately above and below a difference of 0 (green to yellow) for phase.

The least well fit data are at the shortest periods, and there are some areas of correlated misfit, particularly in phase. This may be expected given that the model is trying to fit simultaneously many sites that are widely spread, and the imposed regularisation may systematically over or underestimate the observed data. We note that the Earth’s electrical resistivity is most heterogeneous in the top 5 km (as shown in Figure 3 of the main paper). At longer periods, the fit to both apparent resistivity and phase is uniform and shows no significant spatial bias.

A similar misfit map can be generated for the tipper. Typically, tippers are small at periods < 100 s in sedimentary regions but can be locally large depending on small-scale lateral changes in resistivity that are not resolved by regularised smooth 10 km grids. Thus, Figure S3 only shows normalised tipper magnitude (observed – modelled) fits for periods of 215 and 2154 s. The error floor for the tipper is set at 0.02 so again we would expect the tippers to be fit with the colour bars (green to yellow) above and below 0. There is little evidence for significant spatial correlation in misfit, with just a few isolated differences that are due to noise of local resistivity changes to the specific site.


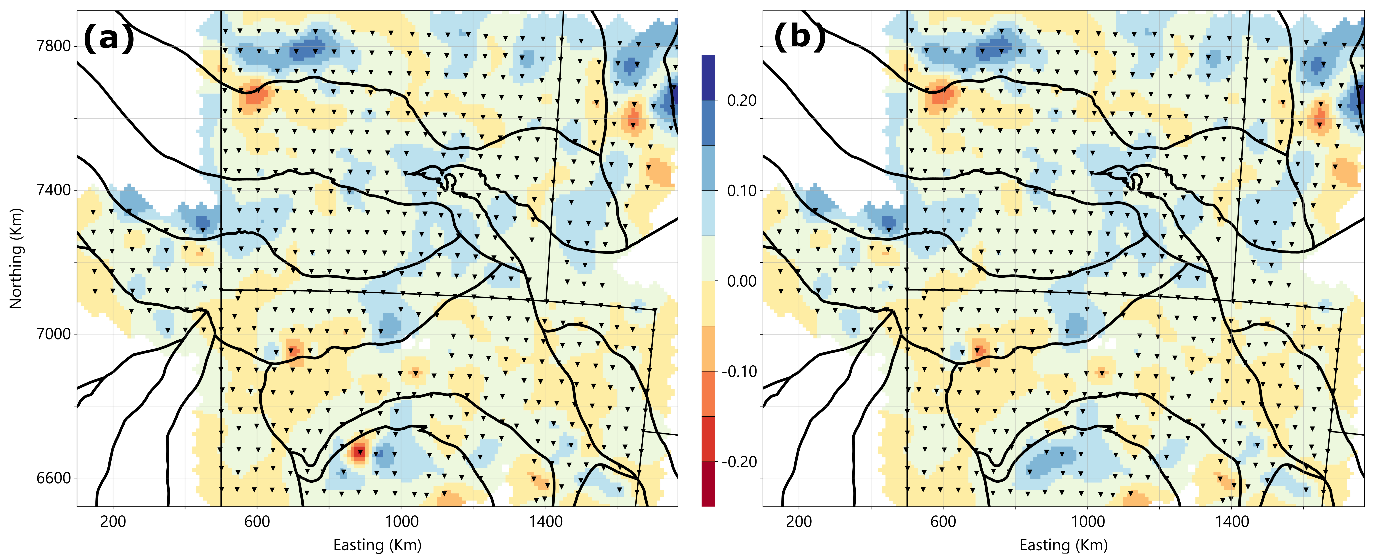


Figure S3: Normalised tipper magnitudes (observed - modelled) for periods of (a) 215 s; (b) 2154 s. Areas are shown in UTM 52S projection. Figures generated using Viridien Geotools V.4.0.3.12574 (<https://www.viridiengroup.com/expertise/multiphysics-imaging/geotools>) and Inkscape V1.2.1 (<https://inkscape.org/cs>).

**Lithosphere-Asthenosphere Conductance**

In the lithospheric mantle, most of the conductance over 100 km thickness is 1-100 S (bulk resistivity of 1000 -100000 Ω.m) which is expected for sub-solidus upper mantle^1^, and with no obvious coherent structure for most of modelled area except for the Mount Isa Province that has higher conductance > 1000 S. The causal mechanism of high conductance for the Mount Isa Province is unknown.


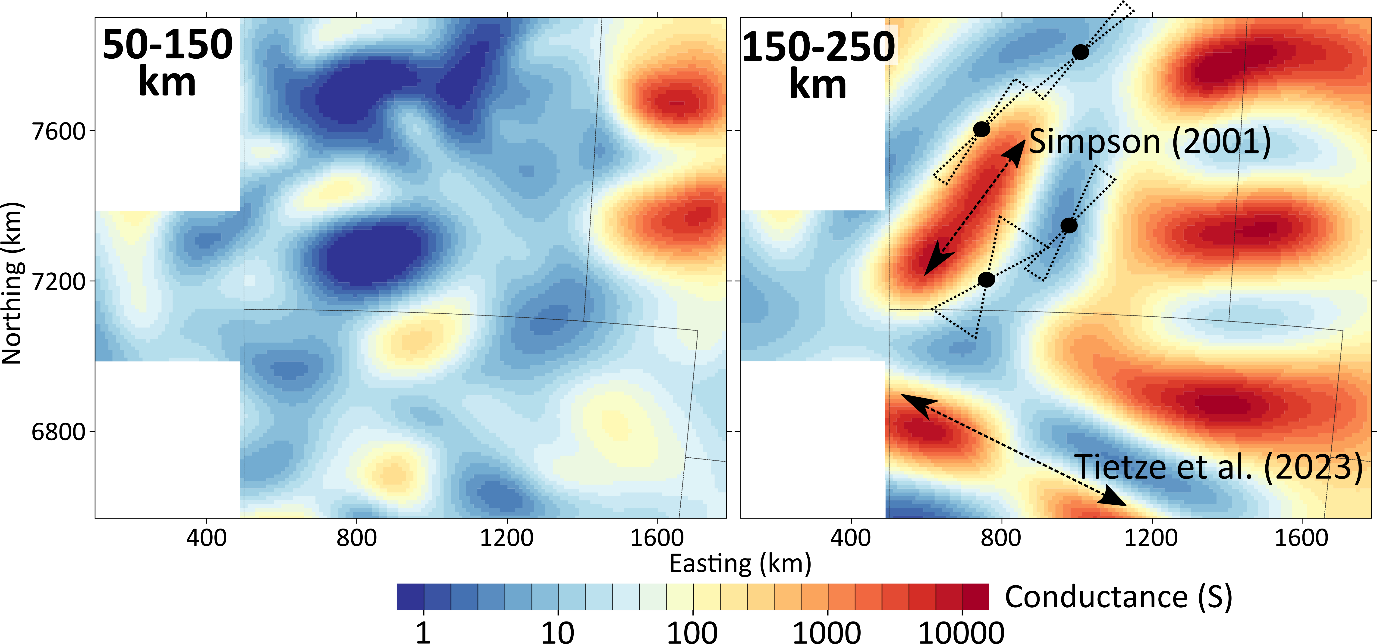


Figure S4: Conductance of the lithosphere from 50-150 km (left), and asthenosphere 150-250 km (right). In the asthenosphere the strike estimates for four Northern Territory MT sites (black circles) are shown with confidence intervals (dashed lines)^2^, and strike direction of the MT Phase tensors for the Gawler Craton is also shown^3^. Areas are shown in UTM 52S projection. Figures generated using Viridien Geotools V.4.0.3.12574 (<https://www.viridiengroup.com/expertise/multiphysics-imaging/geotools>) and Inkscape V1.2.1 (<https://inkscape.org/cs>).

Below 150 km, the conductance of the asthenosphere has a distinct morphology of a series of linear features interspersed between high (>1000 S) and low (<100 S) conductance, spaced about 400 km apart and of a similar strike length of conductor. Observation of significant sub-lithospheric conductors have been observed beneath the Gawler Craton^3^ and the Lachlan Orogen^4^ in southeast Australia. Plotted on this section are the strike angles, with confidence estimates, derived from four long-period MT measurement sites^2^ that were interpreted as being due to mantle anisotropy from olivine alignment in the asthenospheric mantle. The orientation is not parallel to current plate motion of ~28^o^ west of geographic north^5^ and it was argued^2^ that the observed anisotropy of 38^o^ west of geographic north reflected older plate motion orientation. There is good agreement between the strike from the four MT sites and the 3D sub-lithospheric conductor that underlies these sites. Similar asthenospheric anisotropy orientations for central Australia have been determined from SKS and PKS seismic observations^6^. A conductor modelled from AusLAMP data under Gawler Craton was also interpreted as being due to olivine micro-scale anisotropy rather than macro-scale heterogeneity in temperature, composition, but with anisotropy striking in a northwest-southeast direction, aligned with subduction from the south^3^.

The modelled series of macro-conductors below 150 km are the 3D inversion may thus be a manifest of micro-scale anisotropy^7^. It is unclear if this is also the case for the west-east striking conductors on the eastern side of the model and additional AusLAMP sites to the east will be required to further investigate.

**References**

1 Selway, K., Özaydın, S. & Payne, J. Metasomatism and depletion of the southern Gawler Craton from combined mantle xenocryst and AusLAMP magnetotelluric data. *Exploration geophysics (Melbourne)*, 1-15, doi:10.1080/08123985.2023.2282711 (2023).

2 Simpson, F. Resistance to mantle flow inferred from the electromagnetic strike of the Australian upper mantle. *Nature* **412**, 632-635, doi:Doi 10.1038/35088051 (2001).

3 Tietze, K., Thiel, S., Brand, K. & Heinson, G. Comparative 3D inversion of magnetotelluric phase tensors and impedances reveals electrically anisotropic base of Gawler Craton, South Australia. *Exploration Geophysics*, doi:10.1080/08123985.2023.2281615 (2023).

4 Kirkby, A. L. *et al.* Lithospheric architecture of a Phanerozoic orogen from magnetotellurics: AusLAMP in the Tasmanides, southeast Australia. *Tectonophysics* **793**, 228560, doi:10.1016/j.tecto.2020.228560 (2020).

5 Kreemer, C., Blewitt, G. & Klein, E. C. A geodetic plate motion and Global Strain Rate Model. *Geochemistry, Geophysics, Geosystems* **15**, 3849-3889, doi:<https://doi.org/10.1002/2014GC005407> (2014).

6 Eakin, C. M., Flashman, C. & Agrawal, S. Seismic anisotropy beneath Central Australia: A record of ancient lithospheric deformation. *Tectonophysics* **820**, 229123, doi:<https://doi.org/10.1016/j.tecto.2021.229123> (2021).

7 Wannamaker, P. E. Anisotropy Versus Heterogeneity in Continental Solid Earth Electromagnetic Studies: Fundamental Response Characteristics and Implications for Physicochemical State. *Surveys in Geophysics* **26**, 733-765, doi:10.1007/s10712-005-1832-1 (2005).
